# Optimal timing for diagnosis of gestational diabetes as a determinant of pregnancy outcomes: exploring the particularities in a low-income population

**DOI:** 10.20945/2359-4292-2025-0177

**Published:** 2025-09-28

**Authors:** Georgia M. Chichelero, Gabriela J. Hoss, Andrea Auler, Maria L. R. Oppermann, Angela J. Reichelt, Beatriz D. Schaan, Janine Alessi

**Affiliations:** 1 Curso de Ciências Médicas: Endocrinologia, Universidade Federal do Rio Grande do Sul, Porto Alegre, RS, Brasil; 2 Faculdade de Medicina, Universidade de Passo Fundo, Passo Fundo, RS, Brasil; 3 Divisão de Endocrinologia, Hospital de Clínicas de Porto Alegre, Porto Alegre, RS, Brasil; 4 Faculdade de Medicina, Universidade de Santa Cruz do Sul, Santa Cruz do Sul, RS, Brasil; 5 Divisão de Obstetrícia e Ginecologia, Hospital de Clínicas de Porto Alegre, Porto Alegre, RS, Brasil; 6 Faculdade de Medicina, Universidade Federal do Rio Grande do Sul, Porto Alegre, RS, Brasil; 7 Faculdade de Medicina e Programa de Ciências Médicas, Pontifícia Universidade Católica do Rio Grande do Sul, Porto Alegre, RS, Brasil; 8 Divisão de Clínica Médica, Hospital São Lucas, Pontifícia Universidade Católica do Rio Grande do Sul, Porto Alegre, RS, Brasil

**Keywords:** Diabetes, gestational, pregnancy outcome, pregnancy complications, perinatology

## Abstract

**Objective:**

To identify maternal and neonatal outcomes in pregnancies with early versus late gestational diabetes mellitus (GDM) diagnosis, considering healthcare access in a low- to middle-income area of Brazil.

**Subjects and methods:**

This retrospective study included women diagnosed with either early GDM (diagnosed before 20 weeks, based on fasting plasma glucose) or late GDM (diagnosed by 24-28 weeks, via oral glucose tolerance test), according to the IADPSG criteria, who received prenatal care at a hospital in southern Brazil. Maternal outcomes included gestational hypertension, pre-eclampsia, cesarean section or instrumented vaginal delivery, and need for intensive care after birth. Perinatal outcomes were assessed based on the adequacy of birth timing and weight for gestational age, the need for neonatal intensive care, shoulder dystocia or fractures, neonatal hypoglycemia and mortality. Logistic regression was used to adjust for possible confounders, with results presented as odds ratios (OR) and 95% confidence intervals (CI).

**Results:**

A total of 320 women with GDM (mean age 32.9 ± 6.5 years) were included: 164 (51.2%) with early GDM and 156 (48.8%) with late GDM. The primary composite maternal outcome was more frequent in late GDM (43.6% versus 29.3%; OR 1.87; 95% CI 1.15-3.03), as well as perineal laceration (OR 2.45; 95% CI 1.22-4.84). No significant differences were found between groups in the primary composite neonatal outcome, prematurity, or macrosomia rates.

**Conclusion:**

In this low-income population in southern Brazil, early GDM diagnosis led to more prenatal consultations and pharmacological treatment, which may have contributed to reduced adverse maternal outcomes.

## INTRODUCTION

In recent years, alongside the rising prevalence of obesity, there has been a significant increase in the incidence of pregnancies complicated by gestational diabetes mellitus (GDM) (^1^). GDM is associated with a higher risk of adverse maternal and fetal outcomes. In 2015, estimates indicated that 33% of women diagnosed with GDM experienced adverse outcomes (^2^). Maternal complications include cesarean delivery, preeclampsia, and eclampsia, while neonatal complications encompass prematurity, excessive growth (macrosomia and large for gestational age), neonatal hypoglycemia, and admission to intensive care (^2^-^4^).

To identify pregnant women with GDM and implement management strategies earlier to prevent unfavorable outcomes, diagnostic criteria have evolved over time. However, revisions to these criteria have been proposed to reduce the risks of overdiagnosis, which can lead to intensive pregnancy monitoring and increased maternal psychological distress (^5^). Recent studies have compared maternal and neonatal outcomes among women diagnosed with GDM using either strict or flexible criteria. In one study conducted in a middle- to high-income country, the percentage of infants born large for gestational age was similar between the two groups (8.8% and 8.9%, respectively). However, induction of labor, healthcare utilization, pharmacologic treatment, and neonatal hypoglycemia were more prevalent in the group following stricter glycemic criteria (^6^). This scenario may differ in low-income countries with limited access to healthcare services.

Although there is ongoing debate about the ideal cutoff points for diagnosing GDM, the criteria established by the International Association of Diabetes and Pregnancy Study Groups (IADPSG) remain the standard due to their association with improved pregnancy outcomes (^5^). While evidence has highlighted the risks of maternal hyperglycemia during pregnancy, few studies have examined the impact of earlier GDM on reducing maternal and neonatal complications. There is currently no consensus on how the timing of GDM diagnosis during pregnancy affects maternal and neonatal outcomes. An Irish retrospective cohort study demonstrated that women diagnosed with GDM before 24 weeks of gestation had a higher risk of gestational hypertension and postpartum hemorrhage (^7^). Similar findings were reported in a cohort study from Qatar (^8^). However, neither study included low-income populations, and data on outcomes related to GDM timing in economically vulnerable countries remain scarce. Thus, this study aimed to evaluate maternal and neonatal outcomes in pregnancies complicated by GDM based on the timing of diagnosis in a public hospital, employing different diagnostic criteria: fasting blood glucose (FPG) in the first trimester (early GDM) *versus* oral glucose tolerance test (OGTT) at 24-28 weeks of gestation (late GDM).

## SUBJECTS AND METHODS

This was a retrospective study that included pregnant women who delivered their babies between 2017 and 2020 and were diagnosed with GDM while receiving prenatal care at a public tertiary hospital in southern Brazil, located in the city of Porto Alegre, Rio Grande do Sul State, southern Brazil, which provides high-complexity healthcare services. Most patients in this hospital come from low- to middle-income backgrounds. This study was conducted in accordance with the Strenghening the Reporting of Observational Studies in Epidemiology (STROBE) guidelines (^9^).

### Study procedures

#### Identification of participants

To identify potentially eligible participants, an electronic search was conducted to capture all pregnant women diagnosed with GDM who received prenatal care and delivered at the same public hospital between 2017 and 2020. Specified maternal data were then extracted from the electronic medical records by three trained researchers. Birth-related data were also obtained from neonatal records, which were linked to the corresponding maternal records.

Inclusion criteria were based on GDM diagnosis according to the IADPSG/WHO criteria:

1. *Early GDM*: FPG levels greater than 92 mg/dL (≥5.1 mmol/L) and less than 126 mg/dL (<7 mmol/L) at any time before 20 weeks, or
2. *Late GDM:* a 75-g oral glucose tolerance test (OGTT) conducted between 24 and 28 weeks of gestation, with at least one value meeting the following thresholds: FPG greater than 92 mg/dL, one hour greater than or equal to 180 mg/dL (≥10 mmol/L), or two hours greater than or equal to 153 mg/dL (≥8.5 mmol/L).

Data on the dates and methods of diagnosis were extracted from the electronic medical records. Exclusion criteria included women who delivered at another facility (due to missing birth outcome data), those with fewer than one prenatal visit, those with incomplete medical records, and those diagnosed with pregestational diabetes. For participants with multiple pregnancies or deliveries during the study period, only data from the most recent pregnancy were included.

#### Assessment of clinical characteristics

Data regarding demographic characteristics and pregnancy outcomes were retrieved from electronic medical records. A thorough evaluation of clinical characteristics and information related to GDM care was conducted to assess factors that might influence outcomes. Evaluated factors included age, ethnicity, tobacco and substance use, and medication use during pregnancy. Secondary education was considered completed if the participant had at least 11 years of schooling. Pre-gestational body mass index (BMI) was calculated using the Quetelet index, defined as weight in kilograms divided by the square of height in meters (kg/m²). This calculation is based on self-reported pre-gestational weight and height measured at the first prenatal appointment. Gestational weight gain was determined as the difference between the weight measured at the last medical visit and the pre-gestational weight.

#### Study outcomes

##### Maternal outcomes

The primary composite maternal outcome included at least one of the following: polyhydramnios, preeclampsia, gestational hypertension, cesarean delivery, or vaginal delivery requiring episiotomy, instrumental delivery, or resulting in perineal laceration. Polyhydramnios was assessed using the amniotic fluid index, which is the sum of the largest vertical measurements in each of the four uterine quadrants, and was considered present when the index exceeded 18 centimeters from 28 weeks of gestation (^10^). Preeclampsia was defined by the presence of blood pressure measurements of 140/90 mmHg or higher, confirmed on two separate occasions, with significant proteinuria defined as 300 mg or more of protein in a 24-hour urine sample or a urine protein-to-creatinine ratio of 0.3 or higher, systemic involvement, or target organ dysfunction (^11^). Gestational hypertension was diagnosed when hypertension developed after the 20^th^ week of gestation in a woman previously normotensive and in the absence of protein in the urine or other systemic complications. Instrumental delivery included the use of forceps, vacuum extraction, or other facilitators during birth.

Other exploratory outcomes included:

- Number of high-risk prenatal visits, specifically in obstetrics and endocrinology.
- Incidence of hypoglycemia during pregnancy, defined as capillary blood glucose levels less than 70 mg/dL (^1^), as documented in the last available measurement before delivery by the attending clinician.
- Low adherence to the recommended treatment, as assessed based on entries in the medical records and the clinician’s impression recorded at the last prenatal visit.
- Healthcare team’s assessment of glycemic control, categorized as good, fair, or poor, based on the subjective impression noted during the last prenatal visit.
- Admission to the postpartum maternal intensive care unit.

##### Neonatal outcomes

The primary composite neonatal outcome included at least one of the following: macrosomia, prematurity, congenital anomaly, admission to the neonatal intensive care unit, neonatal hypoglycemia, shoulder dystocia, respiratory distress, stillbirth, or neonatal death. Prematurity was defined as birth before 37 weeks of gestation. Macrosomia was identified as birth weight exceeding 4,000 grams. Congenital anomalies potentially related to diabetes were assessed by experts from the Collaborative Latin American Study of Congenital Malformations at *Hospital de Clínicas de Porto Alegre* (^12^) and documented in the newborn’s birth record by the neonatal team. Admission to the neonatal intensive care unit was considered positive if it lasted at least 24 hours. Neonatal hypoglycemia was defined as a serum glucose level of less than 40 mg/dL during the first four hours of life, of less than 45 mg/dL between four and 24 hours of life in term neonates, or if intravenous hypertonic glucose was required. Stillbirth was defined as intrauterine death after 20 weeks of gestation, while neonatal death was defined as death within the first 28 completed days of life (^13^).

### Sample size

The study protocol was designed to assess neonatal outcomes based on the timing of GDM diagnosis. Data from a previous study indicated that the incidence of adverse neonatal outcomes was significantly higher in patients with elevated fasting plasma glucose (early GDM) compared with those diagnosed with a 75-g oral glucose tolerance test (late GDM) (20.4% *versus* 9.3%, P < 0.01) (^14^). Sample size calculation for dichotomous outcomes was based on the expected incidence in each group. It was determined that 320 participants would be necessary to achieve an 80% power, considering a significance level of 0.05.

### Statistical analysis

Analyses were performed using IBM-SPSS v.22 (Chicago, IL, US). Participant characteristics were reported as mean ± standard deviation (SD) when the assumption of normality was not violated; otherwise, data were reported as median ± interquartile range (IQR). Group differences for baseline data were evaluated using unpaired *t*-test and the Mann-Whitney U test for continuous variables, and the chi-square test for categorical variables.

The primary maternal and neonatal composite outcomes were evaluated using Chi Square tests. Second, to assess the impact of various clinical and demographic variables on the primary outcome, logistic regression models were used to adjust outcomes for potential confounders. Odds ratios (OR) and their respective 95% confidence intervals (CI) were calculated using the late GDM diagnostic criteria as the reference group and were adjusted for age, ethnicity, gestational weight gain, and use of metformin during pregnancy, as these variables were identified as possible influencers of neonatal outcomes in pregnancies (^2^,^15^). Sensitivity analyses were performed evaluating the results in different groups by including only singleton pregnancies according to the preplanned protocol.

For this study, statistical significance was set at p < 0.05.

### Ethical aspects

The study was performed in accordance with the Declaration of Helsinki (2004) and followed all relevant guidelines and regulations. It was approved by the Research Ethics Committee of *Hospital de Clínicas de Porto Alegre* (No. 2020-0109) and registered on Plataforma Brasil (CAAE No. 31777520600005327). All authors signed a confidentiality agreement for data use.

## RESULTS

**Figure 1:**
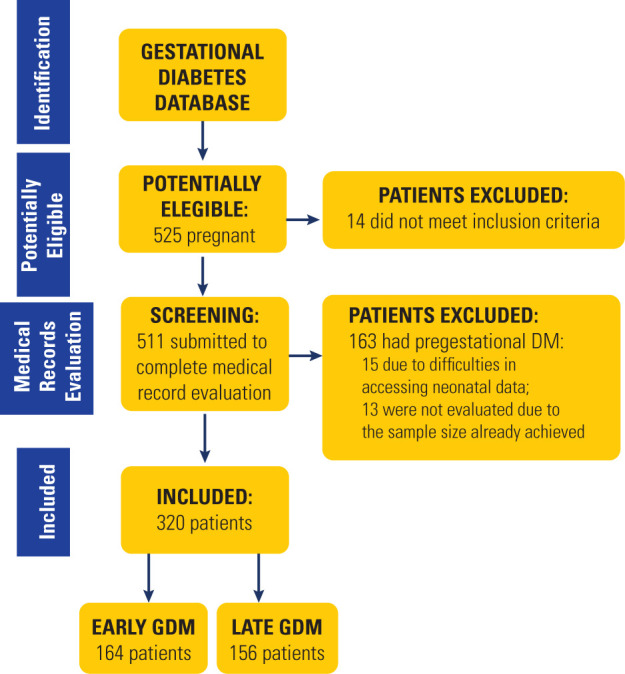
Study flowchart DM: diabetes mellitus; GDM: gestational diabetes mellitus.

 A total of 525 potentially eligible patients were identified, and the selection process was concluded upon reaching the planned sample size. Of the 511 records reviewed, 163 were excluded due to pregestational diabetes, 15 were excluded due to difficulties in accessing data, and 13 were not evaluated as the required sample size had already been achieved (**Figure 1**). In total, 320 pregnant women with GDM were included in the analysis: 164 (51.2%) diagnosed with early GDM (based on FPG measured before 20 weeks) and 156 (48.8%) diagnosed with late GDM (based on the OGTT performed in the second trimester).

### Participants characteristics

Among the 320 participants the mean age was 32.9 ± 6.5 years, with 73.7% identifying as white and 25% being formally married. Overall, 65.6% had completed secondary education, with no difference between the groups (67.9% *versus* 63.4%, p = 0.393). In both groups, the prevalence of smoking, chronic hypertension, family history of diabetes, and personal history of GDM was similar (13.7%). When BMI could be calculated at the beginning of pregnancy, no difference was observed between groups (31.0 ± 8.1 *versus* 32.7 ± 8.2, p = 0.427). However, a higher weight gain during pregnancy was observed in the late GDM group (p = 0.013) (**Table 1**).

**Table 1:** Baseline characteristics of women according to the trimester of GDM diagnosis

	Total (n = 320)	First trimester* Early GDM (n = 164)	Second trimester** Late GDM (n = 156)	P value
Age (years)	32.9 ± 6.5	32.8 ± 6.5	33.0 ± 6.6	0.954
Self-declared skin color (white)	236 (73.7)	123 (75.0)	113 (72.4)	0.539
Marital status (married)	80 (25.0)	41 (25.0)	39 (25.0)	1.000
Scholarity (≥11 years)	210 (65.6)	104 (63.4)	106 (67.9)	0.393
Pre-gestational BMI	31.9 ± 8.2	32.7 ± 8.2	31.0 ± 8.1	0.427
Previous medical history				
Family history of type 2 diabetes	44 (13.7)	22 (13.4)	22 (14.1)	0.858
Current smoker	39 (12.1)	18 (10.9)	21 (13.4)	0.483
Chronic hypertension	63 (19.7)	34 (20.7)	29 (18.5)	0.611
Previous pregnancies	2.7 ± 1.4	2.7 ± 1.3	2.6 ± 1.4	0.564
Previous GDM	44 (13.7)	22 (50)	22 (50)	0.858
Current pregnancy data				
Weight gain (kg)	9.5 (4.9-14.0)	8.0 (3.5-12.5)	10.0 (6.2-14.3)	0.013
Diet only	179 (55.9)	81 (49.3)	98 (62.8)	0.016
Insulin use	39 (12.1)	21 (12.8)	18 (11.5)	0.729
Metformin use	131 (40.9)	78 (47.5)	53 (33.9)	0.013
Urinary tract infection	72 (22.5)	38 (23.1)	34 (21.7)	0.768
Aspirin use	42 (13.1)	25 (15.2)	17 (10.8)	0.250
Folic acid use	78 (24.3)	40 (24.3)	38 (24.3)	0.995
Ferrous sulphate use	112 (35.0)	55 (33.5)	57 (36.5)	0.574

* GDM diagnosed by fasting plasma glucose (FPG).

** GDM diagnosed with the 75-g OGTT. Data are mean ± standard deviation or n (%). Weight gain data: median and interquartile range (IQR). α ≤ 0.05 indicates significant difference. BMI: Body mass index. GDM: Gestational diabetes mellitus. For pre-gestational BMI and weight gain: n = 122 if FPG and n = 130 if OGTT.

A minority of pregnant women in both groups used medications during pregnancy, including ferrous sulfate, folic acid, and aspirin. Regarding GDM management, 55.9% of participants required only dietary measures, which was more frequent among those diagnosed later (second trimester: 62.8% *versus* 49.3%, p = 0.016). In contrast, 40.9% required both dietary measures and metformin, particularly in the group diagnosed earlier (first trimester: 33.9% *versus* 47.5%, p = 0.013). Only 12.1% of participants required insulin during pregnancy.

### Maternal outcomes

The primary composite maternal endpoint occurred more frequently among pregnancies diagnosed with GDM in the second trimester. Specifically, 29.3% of participants in the early GDM group experienced at least one of the adverse events included in the assessment, compared to 43.6% in the late GDM group. This result remained statistically significant even after adjusting for potential confounders (OR 1.87; 95% CI 1.15-3.03) (**Table 2**).

**Table 2:** Maternal outcomes by timing of GDM diagnosis

	Early GDM (n = 164)	Late GDM (n = 156)	P value	OR
**Primary composite maternal outcome** *	48 (29.3)	68 (43.6)	<0.01	**1.87 (1.15-3.03)**
Polyhydramnios	10 (6.2)	5 (3.2)	0.04	1.86 (0.61-5.73)
Preeclampsia	11 (6.7)	19 (12.2)	0.09	0.62 (0.23-1.36)
Gestational hypertension	12 (7.3)	9 (5.8)	0.58	1.35 (0.54-3.36)
Caesarean delivery	108 (65.9)	88 (56.4)	0.08	0.66 (0.41-1.06)
Episiotomy	15 (9.1)	21 (13.5)	0.26	1.49 (0.72-3.07)
Perineal laceration	16 (9.8)	30 (19.2)	0.05	2.43 (1.22-4.84)
Instrumental vaginal delivery	3 (1.8)	3 (1.9)	0.37	0.82 (0.16-4.30)
Postpartum intensive care admission	2 (1.2)	2 (1.3)	0.96	0.89 (0.12-6.69)
Aspects related to diabetes management				
Total consultations in high-risk prenatal care	10.0 (6.3-14.0)	9.5 (7.0-12.0)	0.050	-
Medical impression on glycemic control				-
Optimal	105 (64.0)	114 (73.1)	0.215	-
Average	41 (25.0)	30 (19.2)		-
Suboptimal	18 (11.0)	12 (7.7)		-
Poor adherence	28 (17.1)	22 (14.1)	0.464	-
Hypoglycemia during pregnancy	13 (7.9)	13 (8.3)	0.894	-

Data are presented as median and interquartile range (IQR) or total number (%). Odds ratios (OR) and their respective 95% confidence intervals (CI) were calculated using the late GDM diagnostic criteria as the reference group and were adjusted for age, ethnicity, gestational weight gain, and use of metformin during pregnancy. Early GDM: those diagnosed before 20 weeks of gestation based on fasting plasma glucose level by the fasting plasma glucose (FPG); Late GDM: those identified between 24 and 28 weeks using an 75g oral glucose tolerance test (OGTT).

* The primary composite maternal outcome included at least one of the following: polyhydramnios, preeclampsia, gestational hypertension, cesarean delivery, vaginal delivery requiring episiotomy or instrumental delivery, perineal laceration, or postpartum need for intensive care. α ≤ 0.05 indicates a significant difference.

Regarding individual maternal outcomes, 4.7% of participants experienced polyhydramnios, with no difference between groups. Perineal laceration was more frequent in the late GDM group (OR 2.45; 95% CI 1.22-4.84). Rates of gestational hypertensive disorders were similar between groups, both for preeclampsia (6.7% *versus* 12.2%, p = 0.09) and gestational hypertension (7.3% *versus* 5.8%, p = 0.58). The timing of GDM diagnosis did not influence the mode of delivery, with cesarean section rates of 56.4% in the late GDM group and 65.9% in the early GDM group (p = 0.08).

Concerning diabetes management, the early GDM group tended to require a higher number of prenatal visits (p = 0.05). However, there was no statistically significant difference in the clinical evaluation specifically regarding glycemic control (p = 0.215), underscoring that glycemic management was comparable between these women and those with late GDM; similarly, no difference was found in the history of poor treatment adherence (p = 0.464).

### Neonatal outcomes

Regarding the primary composite neonatal outcome, no differences were observed between women diagnosed with GDM early *versus* late. Specifically, 40.2% of participants in the early GDM group experienced at least one of the assessed events, compared to 43.5% in the late GDM group (p = 0.55). Rates of prematurity (16.0% *versus* 14.3%) and macrosomia (10.7% *versus* 7.5%) were also similar between groups (p = 0.67 and p = 0.31, respectively). Other evaluated outcomes, including congenital anomalies, the need for admission to the intensive care unit, shoulder dystocia, neonatal hypoglycemia, respiratory distress, stillbirth, and neonatal death, also did not differ between groups (**Table 3**).

**Table 3:** Perinatal outcomes in the newborn comparing groups based on the timing of GDM diagnosis

	Early GDM (n = 169^+^)	Late GDM (n = 161^+^)	P value	OR
Primary composite neonatal outcome*	68 (40.2)	70 (43.5)	0.55	0.87 (0.56-1.37)
Macrosomia	18 (10.7)	12 (7.5)	0.31	0.71 (0.32-1.55)
Prematurity	27 (16.0)	23 (14.3)	0.67	0.75 (0.36-1.54)
Congenital anomaly	5 (3.0)	5 (3.1)	0.94	0.95 (0.26-3.47)
Neonatal intensive care	32 (18.9)	41 (25.5)	0.15	1.45 (0.84-2.50)
Neonatal hypoglycemia	6 (3.6)	7 (4.3)	0.71	1.21 (0.39-3.74)
Shoulder dystocia	4 (2.4)	6 (3.7)	0.47	1.53 (0.41-5.69)
Respiratory distress	8 (4.7)	13 (8.1)	0.21	1.69 (0.67-4.28)
Stillborn	1 (0.6)	0 (0.0)	0.33	—
Neonatal death	0 (0.0)	2 (1.2)	0.15	—

Data are presented as total number (%). Odds ratios (OR) and their respective 95% confidence intervals (CI) were calculated using the late GDM diagnostic criteria as the reference group and were adjusted for age, ethnicity, gestational weight gain, and use of metformin during pregnancy.

* The primary composite neonatal outcome included at least one of the following: macrosomia, prematurity, congenital anomaly, neonatal intensive care, neonatal hypoglycemia, shoulder dystocia, respiratory distress, stillborn or neonatal death. ^+^n = 330, considering ten twin pregnancies. α ≤ 0.05 indicates a significant difference.

## DISCUSSION

This study aimed to explore the timing of the diagnosis of GDM as a determinant of pregnancy outcomes in a low-income population. Our results indicated that an early diagnosis of GDM is associated with fewer adverse maternal outcomes, although it is not associated with adverse neonatal outcomes. In pregnancies with late GDM diagnosis, the incidence of composite maternal outcomes was 87% higher, with perineal lacerations occurring 2.4 times more frequently compared to early diagnosed GDM, even after adjusting for glycemic control and other potential confounders. Rates of prematurity and macrosomia were similar between groups.

There are ongoing controversies in the literature regarding the impact of early diagnosis and treatment of GDM on pregnancy outcomes. On the one hand, a precocious diagnosis would be beneficial because it could enable behavioral interventions and ensure that these patients receive appropriate prenatal care earlier. On the other hand, it may lead to disproportionate interventions and negative outcomes resulting from overtreatment. Beyond the difference in the timing of GDM diagnosis, the diagnostic methods used – FPG for early GDM diagnosis and OGTT for late GDM diagnosis – also vary, resulting in differences in diagnostic accuracy. This factor should likewise be considered when assessing maternal and neonatal outcomes.

Previous studies conducted in high- and middle-income settings have not consistently shown significant differences in maternal-fetal outcomes with early GDM diagnosis and treatment (^16^,^17^). A pilot trial indicated increased neonatal intensive care unit admissions due to higher rates of small-for-gestational-age infants, raising questions about the benefits of early hyperglycemia treatment and its potential impact on fetal nutrition (^18^). Another retrospective study compared women with GDM who received early treatment (before 22 weeks) to a group that had hyperglycemia in the first trimester but only received treatment if confirmed by an OGTT after 22 weeks. This study found that early intervention reduced adverse maternal outcomes such as preeclampsia and was associated with less maternal weight gain (^19^). In the context of low-income countries or economically vulnerable regions, challenges in accessing healthcare may persist. Therefore, early GDM diagnosis can help allocate resources necessary for proper monitoring and care. Consistent with this, our study demonstrated a reduction in adverse maternal outcomes with early GDM diagnosis. Another cohort study of Brazilian pregnant women also found no differences in neonatal outcomes between early and late GDM diagnosis (^20^). Additionally, we found no increase in adverse neonatal outcomes potentially attributable to overtreatment, in contrast with findings from studies conducted in settings with more readily available therapeutic resources.

Earlier detection of GDM is often associated with a more unfavorable metabolic profile, and the timing of diagnosis influences the type of treatment employed. In our study, early diagnosis of GDM was associated with a greater likelihood of metformin use, while women diagnosed with GDM later relied their treatment more on dietary management, with no differences in other treatment modalities, such as insulin. Additionally, there was a trend toward a higher number of prenatal care visits when the GDM diagnosis was made earlier, reflecting increased concern and closer clinical monitoring within this group. This greater frequency of appointments may have contributed to improved maternal glycemic control. We speculate that the need for more medical treatment and more frequent visits among those diagnosed earlier with GDM suggests that these women may have faced greater challenges in managing their diabetes. Additionally, they likely benefited from more intensive prenatal monitoring, which facilitated the implementation of appropriate therapeutic measures that contribute to improved maternal outcomes. Initially, one might attribute the differences observed in maternal outcomes to variations in glycemic control between the groups; however, our study did not identify such variations. This reinforces the hypothesis that, in situations of socioeconomic vulnerability, early diagnosis and treatment of GDM may be beneficial.

We can highlight several strengths of our study. Notably, it is one of the first to evaluate the impact of the timing of GDM diagnosis on maternal-neonatal outcomes in a low-income population. Additionally, we included a relatively large number of women with GDM and experienced minimal data loss. Furthermore, some of our findings corroborate those reported by other researchers, which enhances the generalizability of our results. Despite these strengths, this study has some limitations. First, it was conducted at a single center, despite involving a relatively large sample size in accordance with the sample size calculation. Data were retrospectively retrieved from medical records, which could lead to registration and information bias. Additionally, pregestational weight was primarily self-reported, introducing the possibility of recall bias. We also acknowledged that glycemic control was assessed through the subjective judgment of the attending healthcare providers, which may be prone to bias and should be considered when interpreting our findings. Finally, as this is an observational study, the associations identified may not reflect causal relationships, highlighting the need for further research to confirm these findings.

In conclusion, the challenges of managing GDM during pregnancy are even more pronounced when considering socioeconomic difficulties and barriers to accessing healthcare resources. In this context, it is crucial to identify factors that may influence the natural history, treatment, and control of hyperglycemia, thereby positively affecting pregnancy outcomes. In a low-income population in southern Brazil, our findings suggest that pregnant women diagnosed with GDM earlier through FPG tended to receive more medical consultations and pharmacological treatment, which may have contributed to a reduced incidence of adverse maternal outcomes. These findings highlight the relevance of early FPG evaluation as a diagnostic test for GDM, especially in contexts marked by socioeconomic vulnerability and limited access to healthcare, while also indicating the need for further studies to clarify and expand these results.

## Data Availability

datasets related to this article will be available upon request to the corresponding author.
